# The hidden story behind gender differences in familial amyloid polyneuropathy (FAP) ATTRV30M

**DOI:** 10.1186/1750-1172-10-S1-O4

**Published:** 2015-11-02

**Authors:** Diana Santos, Teresa Coelho, Miguel Alves-Ferreira, Jorge Sequeiros, Isabel Alonso, Manuela Grazina, Alda Sousa, Carolina Lemos

**Affiliations:** 1 IBMC and ICBAS, UnIGENe, 4150, Porto, Portugal; 2 Unidade Corino de Andrade, Unidade Corino de Andrade, 4050 - 345, Porto, Potugal; 3 CNC - FMUC, LBG, 3000-354, Porto, Portugal

## Background

Familial amyloid polyneuropathy (FAP ATTRV30M) is an autosomal dominant systemic amyloidosis, due to a point mutation in the transthyretin (TTR) gene (chr18q12.1). The most frequent one, V30M is associated with several clusters. Among Portuguese families, FAP shows a wide variation in in age-at-onset (AO) [19-82 yrs] and this variability is also apparent between generations. Also, significant differences in AO regarding gender are known in Portuguese series, where women were found to have a later-onset than men. Moreover, mother-son pairs showed larger anticipation (> 10 yrs) while the father-daughter pairs only showed residual anticipation. Therefore, to unravel these gender-related differences in AO, we studied three candidate-genes (AR, HSD17B1 and BGN) linked to sex-steroid hormones or X-linked as genetic modifiers of AO. We also evaluated if mitochondrial DNA (mtDNA) copy number is associated with AO.

## Methods

We analysed a DNA sample of 318 Portuguese patients (106 families) corresponding to 152 males and 166 females. Additionally, asymptomatic carriers and non-carriers were also included in the study. Polymorphisms in candidate genes were genotyped by several standard techniques and mtDNA copy number was assessed using appropriate software for analysis.

## Results

Our patients' sample shows a mean AO of around 39 years, but mean AO in males (37.28) is lower than in females (40.52), as already described in the literature. Moreover, we found some polymorphisms significantly associated with AO variation. For the AR gene, in the male group, three polymorphisms were associated with an early AO, while in the female group, four were associated with both an early and later AO. Regarding parental transmission in this gene, for rs5919392, we found that e affected mothers transmitted the T allele more often than expected (which is associated with an early-onset). For HSD17B1 gene, we did not find any significant results. Concerning BGN gene, in the male group no significant results were found associated with AO but, in the female group, one polymorphism was associated with a later AO. Regarding mtDNA copy number, there are significant gender differences when we compared controls and patients groups. Patients present an mtDNA copy number higher than controls. We also found significant differences in the female group when we compared late and early patients.

## Conclusions

This study revealed for the first time the contribution of the AR and BGN genes as AO modifiers both in males and females. Moreover, it was important to show that mtDNA copy number is associated with FAP. Therefore, we showed that FAP expresses differently in males and females. These results are significant to improve clinical management, with important implications in genetic counselling and therapeutic strategies.

